# Direct Gloving vs Hand Hygiene Before Donning Gloves in Adherence to Hospital Infection Control Practices: A Cluster Randomized Clinical Trial

**DOI:** 10.1001/jamanetworkopen.2023.36758

**Published:** 2023-10-26

**Authors:** Kerri A. Thom, Clare Rock, Gwen L. Robinson, Heather Schacht Reisinger, Jure Baloh, Shanshan Li, Daniel J. Diekema, Loreen A. Herwaldt, J. Kristie Johnson, Anthony D. Harris, Eli N. Perencevich

**Affiliations:** 1 Department of Epidemiology, University of Maryland School of Medicine, Baltimore, Maryland; 2 Division of Infectious Diseases, Department of Medicine, Johns Hopkins School of Medicine, Baltimore, Maryland; 3 University of Iowa Carver College of Medicine, Iowa City, Iowa; 4 Department of Health Policy and Management, University of Arkansas for Medical Sciences, Little Rock; 5 MassMutual Data Science, Springfield, Massachusetts

## Abstract

**Importance:**

Current guidelines require hand hygiene before donning nonsterile gloves, but evidence to support this requirement is lacking.

**Objective:**

To evaluate the effectiveness of a direct-gloving policy on adherence to infection prevention practices in a hospital setting.

**Design, Setting, and Participants:**

This mixed-method, multicenter, cluster randomized clinical trial was conducted at 4 academic centers in Baltimore, Maryland, or Iowa City, Iowa, from January 1, 2016, to November 30, 2017. Data analysis was completed April 25, 2019. Participants were 3790 health care personnel (HCP) across 13 hospital units.

**Intervention:**

Hospital units were randomly assigned to direct gloving, with hand hygiene not required before donning gloves (intervention), or to usual care (hand hygiene before donning nonsterile gloves).

**Main Outcomes and Measures:**

The primary outcome was adherence to the expected practice at room entry and exit. A random sample of HCPs’ gloved hands were imprinted on agar plates at entry to contact precautions rooms. The intention-to-treat approach was followed, and all analyses were conducted at the level of the participating unit. Primary and secondary outcomes between treatment groups were assessed using generalized estimating equations with an unstructured working correlation matrix to adjust for clustering; multivariate analysis using generalized estimating equations was conducted to adjust for covariates, including baseline adherence.

**Results:**

In total, 13 hospital units participated in the trial, and 3790 HCP were observed. Adherence to expected practice was greater in the 6 units with the direct-gloving intervention than in the 7 usual care units (1297 of 1491 [87%] vs 954 of 2299 [41%]; *P* < .001) even when controlling for baseline hand hygiene rates, unit type, and universal gloving policies (risk ratio [RR], 1.76; 95% CI, 1.58-1.97). Glove use on entry to contact precautions rooms was also higher in the direct-gloving units (1297 of 1491 [87%] vs 1530 of 2299 [67%]; *P* = .008. The intervention had no effect on hand hygiene adherence measured at entry to non–contact precautions rooms (951 of 1315 [72%] for usual care vs 1111 of 1688 [66%] for direct gloving; RR, 1.00 [95% CI, 0.91-1.10]) or at room exit (1587 of 1897 [84%] for usual care vs 1525 of 1785 [85%] for direct gloving; RR, 0.98 [95% CI, 0.91-1.07]). The intervention was associated with increased total bacteria colony counts (adjusted incidence RR, 7.13; 95% CI, 3.95-12.85) and greater detection of pathogenic bacteria (adjusted incidence RR, 10.18; 95% CI, 2.13-44.94) on gloves in the emergency department and reduced colony counts in pediatrics units (adjusted incidence RR, 0.34; 95% CI, 0.19-0.63), with no change in either total colony count (RR, 0.87 [95% CI, 0.60 to 1.25] for adult intensive care unit; RR, 0.59 [95% CI, 0.31-1.10] for hemodialysis unit) or presence of pathogenic bacteria (RR, 0.93 [95% CI, 0.40-2.14] for adult intensive care unit; RR, 0.55 [95% CI, 0.15-2.04] for hemodialysis unit) in the other units.

**Conclusions and Relevance:**

Current guidelines require hand hygiene before donning nonsterile gloves, but evidence to support this requirement is lacking. The findings from this cluster randomized clinical trial indicate that a direct-gloving strategy without prior hand hygiene should be considered by health care facilities.

**Trial Registration:**

ClinicalTrials.gov Identifier: NCT03119389

## Key Points

**Question:**

Does a strategy of direct gloving compared with performing hand hygiene before donning nonsterile gloves influence adherence to infection prevention practices among health care personnel?

**Findings:**

This mixed-method, multicenter, cluster randomized trial including 3790 health care personnel across 13 hospital units of 4 academic centers demonstrated a statistically significant 46% increase in adherence to a direct-gloving strategy vs usual care of hand hygiene before donning gloves (87% vs 41% adherence).

**Meaning:**

These results suggest that a policy endorsing direct gloving may increase adherence to expected infection prevention practices and overall glove use in many hospital settings.

## Teaser

This cluster randomized clinical trial compares the effectiveness of a direct-gloving policy with the usual practice of hand hygiene before donning nonsterile gloves on adherence of health care personnel to infection prevention practices across several hospital units.

## Introduction

Hand hygiene is the cornerstone of infection prevention, but a prior comprehensive review found that typical adherence in health care settings is only 40%.^1^ Insufficient time, high workload, and understaffing are important barriers. Glove use, which is common and increasing, is another major barrier.^1,2^ Hand hygiene before donning gloves is a current standard, but this practice can delay care delivery because health care personnel (HCP) must wait for their hands to dry before donning gloves, which could contribute to reduced adherence. This delay would be worthwhile if hand hygiene before donning gloves improved safety, yet studies have demonstrated no difference in glove contamination when nonsterile gloves are donned directly vs after performing hand hygiene.^3,4^ The need for hand hygiene before donning gloves remains an unresolved issue, as current guidelines present conflicting recommendations for hand hygiene.^5,6^ New strategies are needed that safely reduce time and improve efficiency of these critical infection prevention efforts, particularly in settings in which glove use is required and most beneficial (eg, contact precautions). We performed a cluster randomized trial to evaluate the benefits of a protocol that did not require hand hygiene before donning nonsterile gloves (ie, direct gloving). We hypothesized that a direct-gloving strategy compared with usual care would lead to increased adherence with expected hand hygiene and glove use (based on study assignment) and would have no effect on the bacterial contamination of HCPs’ hands.

## Methods

### Study Setting and Participants

This cluster randomized clinical trial was performed from January 1, 2016, to November 30, 2017, in multiple clinical settings (ie, adult intensive care units [ICUs], general pediatrics wards, emergency departments (EDs), and inpatient hemodialysis units at 4 academic health care centers: University of Maryland Medical Center, R. Adams Cowley Shock Trauma Center, and Johns Hopkins Hospital in Baltimore, Maryland, and the University of Iowa Hospitals and Clinics in Iowa City, Iowa. Participants were HCP who delivered care to patients in enrolled study units. This study was approved by the institutional review boards at the University of Maryland, The Johns Hopkins University, and the University of Iowa, which waived the requirement to obtain informed consent for the primary study because no individual health care workers were enrolled. This report adheres to the Consolidated Standards of Reporting Trials (CONSORT) reporting guideline for randomized clinical trials. The full trial protocol is given in Supplement 1.

### Study Design

We performed a mixed-method study including a cluster randomized trial to evaluate the effectiveness of a direct-gloving strategy in which each participating unit was assigned to either usual care (following the hospital’s policy to perform hand hygiene before donning nonsterile gloves) or the direct-gloving intervention (unit-based policies were updated to allow HCP to don nonsterile gloves without first performing hand hygiene). We stratified the randomization of participating units by unit type with the intention of selecting 1 unit from each unit type at each hospital. In addition, to account for differences in baseline adherence to infection prevention practices, including hand hygiene and glove use, we observed baseline rates for 6 months across all 33 available units at participating health care centers before we selected and randomly assigned the units to the intervention (direct-gloving units) or to usual care (usual care units). When possible, units with similar baseline adherence where selected for randomization. After baseline data collection and final unit selection, we performed a 3-month wash-in period during which HCP were educated to follow the policy to which their units were assigned. We implemented the intervention during the final 12th month of the study.

### Intervention

We randomly assigned participating units to either usual care or direct gloving. Randomization was stratified by unit type by the study statistician (S.L.). Immediately before the intervention period, we delivered identical education regarding hand hygiene and glove use to HCP in all participating units via face-to-face meetings, emails, and printed materials. We instructed HCP on usual care units to perform hand hygiene before donning nonsterile gloves, and we instructed HCP on direct-gloving units that hand hygiene was not necessary before donning nonsterile gloves.

### Outcomes

The primary outcome was a composite of adherence to the expected practices of hand hygiene and glove use on entry to contact precautions rooms^7^ based on treatment assignment. We defined assignment adherence for the usual care group as performing hand hygiene and then donning nonsterile gloves before room entry, and assignment adherence for the direct-gloving group as donning nonsterile gloves, regardless of whether HCP performed hand hygiene. Secondary outcomes included rates of adherence to glove use on entry to contact precautions rooms and the balancing measures of overall adherence to hand hygiene on entry and exit to any room type.

### Data Collection

Study staff at each site used a standardized hand hygiene data collection tool to capture hand hygiene and glove use on room entry and exit. Study staff used this form when observing HCP adherence to expected hand hygiene and glove use practice in each of the potentially eligible units (baseline period) and participating units (intervention period). A minimum number of observations was required for each study unit per month, and a randomization tool was used to balance the order of units and the time of day observed at each site. Study staff performing data collection moved to a new location or unit after 15 minutes to avoid the Hawthorne observation effect.^8,9^ Regular study staff meetings reinforced standardized data collection practices.

### Sample Size and Effect Size

We estimated an effect size of approximately 30% based on prior studies that reported mean hand hygiene compliance rates of 40% (and lower when glove use is indicated^1^) and 70% compliance with glove use when indicated.^10^ Assuming 500 observations per unit, an α of .05, and an interclass coefficient of 0.001,^11^ we estimated we would require inclusion of 4 patient care areas (2 experimental and 2 usual care) to achieve a power greater than 90% to detect an effect size of 30% for the primary outcome. To achieve generalizability and feasibility of data collection, we planned to enroll 14 units in total in the study.

### Validation of Safety and Efficacy

The safety and efficacy of a direct-gloving strategy has been previously demonstrated.^4^ We validated those prior findings by sampling gloved hands of HCP immediately after they donned nonsterile gloves before they entered patient rooms in which glove use was expected (eg, contact precaution rooms) among both usual care and direct-gloving units. After verbal consent, HCP imprinted their nondominant gloved hands onto agar plates. We used previously described methods to determine the total bacterial colony counts and the presence of pathogenic bacteria.^4^

### Qualitative Assessment

To assess potential facilitators and barriers to a direct-gloving strategy, we conducted a qualitative evaluation of HCP perceptions in eligible units. The qualitative data collection and analysis for this study are described in detail elsewhere.^12^ We conducted semistructured interviews among a purposeful sample of HCP, nurses, and nursing assistants from direct-gloving and usual care units at each of the 4 sites. The qualitative analysts (H.S.R., J.B.) developed a codebook consisting of inductive and deductive themes.^13^ Data were coded and thematically analyzed in MAXQDA, 2018 (VERBI Software), a qualitative data management and analysis software program.

### Statistical Analysis

We followed the intention-to-treat approach and conducted all analyses at the level of the participating unit. We compared primary and secondary outcomes between treatment groups using a generalized estimating equations approach with an unstructured working correlation matrix to adjust for clustering. We performed multivariate analysis using generalized estimating equations to adjust for covariates, including baseline adherence. Analyses were completed using R, version 3.5.3 (R Foundation for Statistical Computing) on April 25, 2019. Statistical significance was defined as a 2-sided *P* < .05 or 95% CI excluding 1.

## Results

### Baseline Hand Hygiene and Qualitative Assessment

Study staff collected data from all 33 available units—20 ICUs, 7 general pediatrics wards, 3 hemodialysis units, and 3 EDs—at the 4 participating centers during the 6-month baseline period (Figure). In total, 4319 HCP were observed at entry to contact precautions rooms. Adherence to expected practice of hand hygiene followed by glove use was 1522 hand hygiene events of 4319 hand hygiene opportunities (35%; 95% CI, 33%-37%), ranging from a low of 8% in EDs to a high of 63% in hemodialysis units. An additional 2045 of 4319 (47%; 95% CI, 45%-50%) observed HCP donned gloves directly without first performing hand hygiene.

**Figure.**
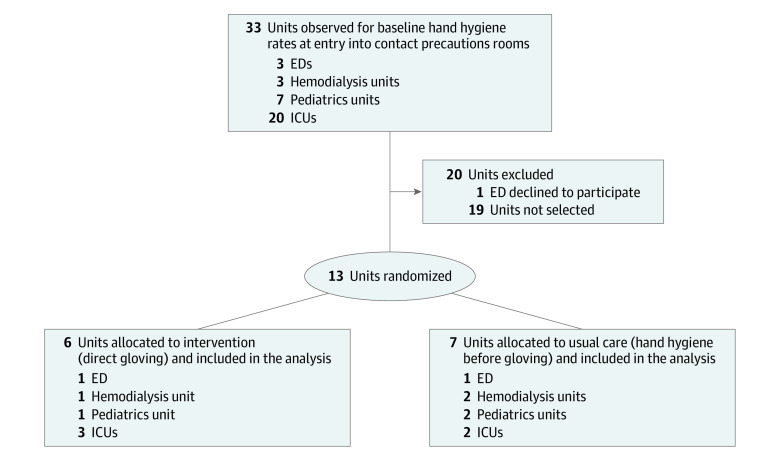
Trial Flow Diagram ED indicates emergency department; ICU, intensive care unit.

We conducted semistructured interviews with 25 HCP (5 physicians, 1 physician assistant, 3 nurse practitioners, 8 nurses, and 8 nursing assistants) across 9 of the 13 randomized units. When asked about the perceived benefits of a direct-gloving strategy, 17 of 25 (68%) perceived potential benefits, including increased efficiency in time (14) and cost, less skin irritation (6), improved adherence to expectations (4), and improved staff satisfaction (3); responses were not mutually exclusive and were often discussed as a cluster of perceived benefits. Fifteen of 25 participants (60%) did not perceive risks or concerns associated with a direct-gloving strategy if proven safe; participants who did cite risks related them to undermining habits (6) and uncertainty about contamination (4), which the participants thought could compromise patient or HCP safety. One participant thought this practice could negatively affect patient perceptions, and 2 participants felt uncomfortable with this practice. The eTable in Supplement 2 provides example quotations from these thematic codes.

### Intervention

Thirteen units were randomly assigned to either usual care (7) or direct gloving (6); 1 ED declined to participate. Table 1 provides the baseline characteristics of the participating units. In total, 3790 HCP were observed at entry to contact precautions rooms. Adherence to expected practice was greater in the direct-gloving units (1297 of 1491 [87%]) than in the usual care units (954 of 2299 [41%]; *P* < .001). This association remained even after controlling for baseline hand hygiene rate and unit characteristics, such as unit type and universal gloving policies (risk ratio [RR], 1.76; 95% CI, 1.58-1.97) (Table 2). Glove use on entry to contact precautions rooms was also greater in the direct-gloving units (1297 of 1491 [87%] vs 1530 of 2299 [67%]; *P* = .008 even after controlling for baseline hand hygiene rates (RR, 1.14; 95% CI, 1.03-1.26). The percentage of HCP who were not adherent to either hand hygiene or glove use on entry to contact precautions rooms was lower in the direct-gloving units (107 of 1491 [7%]) than in usual care units (413 of 2299 [18%]; *P* = .009).

**Table 1.** Baseline Characteristics of 13 Participating Units

Unit	Usual care, No. of total participants (%)	Direct gloving, No. of total participants (%)
Total units, No.	7	6
Unit type, No.		
Adult intensive care	2	3
General pediatrics	2	1
Hemodialysis	2	1
Emergency department	1	1
Universal gloving required, No.^a^	2	1
Baseline adherence, No. adherent of total No. observed (%)^b^	282 of 927 (30)	236 of 633 (37)
Adult intensive care	76 of 217 (35)	115 of 332 (35)
General pediatrics	64 of 300 (21)	30 of 151 (20)
Hemodialysis	168 of 260 (65)	91 of 150 (61)
Emergency department^c^	12 of 150 (8)	NA

Abbreviation: NA, not applicable.

^a^ Three participating units (2 usual care and 1 direct-gloving unit) required universal gloving for entry into any patient room during the study period as part of hospital infection prevention practices.

^b^ Health care personnel were considered adherent in the baseline period if they were compliant with expected practice of hand hygiene followed by glove use before entry into a contact precautions room.

^c^ Of the 3 eligible emergency departments, 1 declined to participate; study staff did not observe any health care personnel enter contact precautions rooms in 1 of the 2 remaining emergency departments because that unit infrequently used contact precautions rooms.

**Table 2.** Multivariable Regression Models of Adherence to Hand Hygiene and Glove Use

Variable	Adherence to expected practice on room entry^a^		Adherence to glove use on room entry^b^		Adherence to hand hygiene on entry into non–contact precautions room^b^		Adherence to hand hygiene on exit from any room type^b^	
	Risk ratio (95% CI)	*P* value	Risk ratio (95% CI)	*P* value	Risk ratio (95% CI)	*P* value	Risk ratio (95% CI)	*P* value
Direct-gloving intervention^c^	1.76 (1.58-1.97)	<.001	1.14 (1.03-1.26)	.01	1.00 (0.91-1.10)	.94	0.98 (0.91-1.07)	.71
Baseline hand hygiene rate^d^	2.30 (1.11 to 4.80)	.03	3.24 (1.75-6.03)	<.001	1.73 (1.10-2.75)	.02	ND	ND
Universal gloving unit^d^	0.78 (0.65-0.94)	.01	ND	ND	ND	ND	ND	ND
Adult intensive care^d^	ND	ND	ND	ND	1.41 (1.19-1.66)	<.001	ND	ND
General pediatrics^d^	1.68 (1.23-2.30)	.001	ND	ND	1.53 (1.29-1.82)	<.001	ND	ND

Abbreviation: ND, not determined.

^a^ Primary outcome; health care personnel were considered adherent in the baseline period if they were compliant with expected practice of hand hygiene followed by glove use before entry into a contact precautions room among 13 participating units.

^b^ Secondary outcome.

^c^ Exposure variable.

^d^ Covariate.

The direct-gloving strategy had no independent effect either on hand hygiene adherence measured at entry to non–contact precautions rooms (951 of 1315 [72%] for usual care vs 1111 of 1688 [66%] for direct gloving; RR, 1.00 [95% CI, 0.91-1.10]) or on hand hygiene adherence at room exit from any room type (1587 of 1897 [84%] for usual care vs 1525 of 1785 [85%] for direct gloving; RR, 0.98 [95% CI, 0.91-1.07]) even after controlling for factors such as baseline hand hygiene rates, study month, or unit type.

### Safety and Efficacy Validation

We observed 2383 HCP at random on entry to contact precautions rooms, 1194 in direct-gloving units and 1189 in usual care units, and we sampled their gloves for bacteria before room entry. Pathogens were identified in 49 of 1194 (4%) samples obtained from the direct-gloving units, with a mean (SD) total bacterial colony count of 16.3 (45.9) colony-forming units (CFUs), whereas 28 of 1189 (2%) samples obtained in usual care units had pathogens identified, with a mean of 9.5 (33.2) CFUs. Overall, pathogenic bacteria among the different types of units ranged from 1% to 4% and overall bacterial burden ranged from 5.3 to 12.1 CFUs. In the pediatrics unit, the direct-gloving policy had a protective effect on total colony counts (adjusted incidence RR, 0.34; 95% CI, 0.19-0.63). However, the ED had a higher prevalence of pathogenic bacteria (13%) and greater bioburden (52.8 CFUs) in the direct-gloving units (Table 3). The adjusted incidence RRs for the ED also showed a significantly greater incidence of pathogenic bacteria detected on gloved hands (10.18; 95% CI, 2.31-44.94) and higher total colony counts (7.13; 95% CI, 3.95-12.85) in direct-gloving units compared with usual care units. Unit type was an effect modifier, and thus results are presented stratified by unit type (Table 3 and Table 4).

**Table 3.** Detection of Bacteria on Gloves at Entry to Contact Precautions Rooms

Unit	Participants, No.		Total colony count, mean (SD) CFUs		Detection of pathogenic bacteria, No. of total participants (%)	
	Direct gloving	Usual care	Direct gloving	Usual care	Direct gloving	Usual care
Overall	1194	1189	16.3 (45.9)	9.5 (33.2)	49 of 1194 (4)	28 of 1189 (2)
Adult intensive care	599	331	12.1 (34.5)	10.4 (35.9)	18 of 599 (3)	9 of 331 (3)
General pediatrics	220	330	5.8 (10.0)	9.2 (31.5)	3 of 220 (1)	5 of 330 (2)
Hemodialysis	186	363	5.3 (10.8)	10.6 (38.6)	3 of 186 (2)	12 of 363 (3)
Emergency department	189	165	52.8 (88.0)	5.9 (11.6)	25 of 189 (13)	2 of 165 (1)

Abbreviation: CFUs, colony-forming units.

**Table 4.** Adjusted Incidence Risk Ratios for the Detection of Bacteria on Gloves in Direct-Gloving and Usual Care Units, by Total Colony Counts and Detection of Potential Pathogens^a^

Variable	Total colony counts^b^		Detection of pathogens^c^	
	Adjusted incidence risk ratio (95% CI)	*P* value	Adjusted incidence risk ratio (95% CI)	*P* value
Direct-gloving intervention (vs control), by unit type^d^				
Adult intensive care	0.87 (0.60-1.25)	.45	0.93 (0.40-2.14)	.86
General pediatrics	0.34 (0.19-0.63)	<.001	0.59 (0.13-2.60)	.49
Hemodialysis	0.59 (0.31-1.10)	.10	0.55 (0.15-2.04)	.37
Emergency department	7.13 (3.95-12.85)	<.001	10.18 (2.31-44.94)	.002
Health care personnel type (vs nurse)				
Physician	0.64 (0.43-0.92)	.02	0.26 (0.06-0.75)	.03
Other	1.33 (1.02-1.73)	.03	1.37 (0.74-2.48)	.30
Time since last hand hygiene (vs >30 min Prior)				
<1 min prior	0.15 (0.09-0.24)	<.001	0.24 (0.06-1.18)	.05
1 to <5 min Prior	0.30 (0.19-0.49)	<.001	0.41 (0.11-2.07)	.23
5-30 min Prior	0.51 (0.32-0.83)	.005	0.73 (0.19-3.69)	.67

^a^ Based on Poisson regression with unit type (4 levels), intervention group (2 levels) and an interaction term between the 2 as the risk factors, while adjusting for the control variables listed in the table; sample size is 2383.

^b^ *P* < .001 for the interaction term of unit type by intervention group for the outcome total colony counts.

^c^ *P* = .11 for the interaction term of unit type by intervention group for the outcome detection of pathogenic bacteria.

^d^ Unit type was found to be an effect modifier; thus, results are presented stratified by unit type.

When compared with nurses, physicians had lower bacterial contamination of gloves (adjusted incidence RR, 0.64; 95% CI, 0.43-0.92), and other HCP, such as respiratory therapists and physical therapists, had higher bacterial contamination of gloves (adjusted incidence RR, 1.33; 95% CI, 1.02-1.73) (Table 4). Longer time between the last hand hygiene event and sampling was associated with higher total colony counts (mean [SD] >30 minutes, 32.7 [76.9] CFUs; 5-30 minutes, 18.8 [50.4] CFUs; 1-<5 minutes, 11.7 [36.8] CFUs; and <1 minute, 4.9 [15.8] CFUs) and more frequent detection of bacteria on gloves.

The ED had the lowest overall hand hygiene rates and glove use compared with other units. Specifically, the ED had the lowest hand hygiene rate (12 of 150 [8%]) before glove use compared with other unit types and had the lowest hand hygiene adherence at entry to non–contact precautions rooms (433 of 923 [47%] vs 1629 of 2080 [78%] and at exit from any room (379 of 537 [71%] vs 2733 of 3145 [87%]). In the ED, HCP entering non–contact precautions rooms performed hand hygiene or wore gloves less often than HCP in other units (297 of 923 [32%] vs 288 of 2080 [14%]). More HCP (324 of 354 [92%]) sampled in the ED performed hand hygiene longer than 1 minute before sampling compared with other units (1626 of 2029 [80%]). Glove use was also lower in the ED for contact precautions rooms (261 of 397 [66%]) compared with all other units (3841 of 5221 [74%]).

## Discussion

This cluster randomized clinical trial demonstrated that a policy endorsing a direct-gloving strategy compared with the current strategy requiring hand hygiene before glove use led to improved adherence with expected practices and increased overall glove use, was accepted by HCP, and did not increase bacterial contamination of gloves in most clinical areas, except where hand hygiene rates were low (ie, ED). Hands, including gloved hands, are the most important contributors to pathogen transfer in health care, resulting in pathogen spread and health care-associated infection.^14^ The World Health Organization guidelines on hand hygiene in health care^5^ recommend 5 key moments for hand hygiene and include a recommendation to perform hand hygiene before donning gloves. Yet barriers continue to limit adherence, including time taken to let hands dry after alcohol- or water-based hand hygiene. Furthermore, adherence with expected hand hygiene on room entry has been negatively associated with glove use.^1,2^ Evidenced-based strategies are needed that improve practice by helping HCP efficiently integrate infection prevention practices into their work flow. Prior studies have shown direct gloving to be safe.^4^

A rigorous approach to evidenced-based guidelines is needed to increase acceptance and adherence while improving efficiency and decreasing unnecessary demands on HCPs’ time. We found poor adherence with the expectation of hand hygiene before nonsterile glove use in the baseline period of this study at 35% overall, ranging from a low of 8% in EDs to a high of 63% in hemodialysis units, rates that are similar to prior reports.^2,15,16,17,18^ Demands on time, such as high work load and understaffing, are cited as important barriers, and these issues are compounded when gloves are worn. Prior studies have found that performing hand hygiene before donning gloves took an additional 32 to 46 seconds compared with directly donning gloves, presumably because HCP had to wait for their hands to dry before donning new gloves.^4,17^ Additionally, at least 1 report indicates that HCP may not believe that hand hygiene before donning nonsterile gloves is a scientifically necessary step^12^ but is rather a matter of guideline adherence. This belief is perhaps due to the disconnect between guideline recommendations and underlying supporting evidence.

In the present study, the unit-based policy of the intervention allowing direct gloving when nonsterile gloves were worn led to 46% improved adherence to expectations. The intervention was also associated with a positive effect on glove use, leading to more appropriate use of gloves when expected for entry into contact precautions rooms or universal gloving areas. Additionally, there were no untoward effects on balancing measures, such as hand hygiene at room entry to non–contact precautions rooms or at room exit, despite potential concerns outlined in the semistructured interviews of HCP. These results indicate that an evidenced-based policy for direct gloving may have broad advantages with adherence to infection prevention practices.

In a prior randomized trial, Rock et al^4^ showed that a direct-gloving strategy compared with the usual expected practice of hand hygiene before donning nonsterile gloves had no significant difference on total bacterial colony counts (mean, 8.1 vs 6.9 CFUs; *P* = .52) and a low overall prevalence of potential pathogenic organisms on gloves in both groups. In the present study, we found similar results in all settings, with the exception of the ED. The detection of pathogenic bacteria among the adult ICUs, general pediatrics wards, and hemodialysis units ranged from 1% to 4%, and overall bacterial burden ranged from 5.3 to 12.1 CFUs. Adjusted incidence RRs for both outcomes assessing prevalence of potential pathogens and bioburden of gloved hands supported the prior findings of no difference between direct gloving and usual care. There appeared to be a protective effect of a direct-gloving policy, particularly in the general pediatrics ward, on total colony counts (adjusted incidence RR, 0.34; 95% CI, 0.19-0.63).

By contrast, we found high rates of contamination in the ED, particularly units that adopted the direct-gloving policy. The prevalence of pathogenic bacteria on gloved hands in EDs with direct gloving was 13% and associated with a bioburden greater than seen elsewhere in the study of 52.8 CFUs (Table 3 and Table 4). Adjusted incidence RRs showed a significantly greater incidence of pathogenic bacteria detected on gloved hands (10.18; 95% CI, 2.31-44.94) and higher total colony count (7.13; 95% CI, 3.95-12.85) when comparing observations from the direct-gloving units to those from the usual care. While we are unable to explain this finding, we have some hypotheses regarding possible contributing factors. First, overall hand hygiene rates were lower in the ED than in other units (Table 1), which is consistent with published data.^19^ In the baseline period, we noted hand hygiene before glove use was lowest (8%) in the ED. Similarly, hand hygiene adherence at entry to non–contact precautions rooms and at room exit were lower in the ED compared with other unit types (47% vs 78% on entry to non–contact precautions rooms and 71% vs 87% at room exit); furthermore, 32% of HCP who entered non–contact precautions rooms failed to either perform hand hygiene or wear gloves compared with only 14% in other units. Additionally, 92% of HCP sampled in the ED performed hand hygiene more than 1 minute before sampling compared with 80% in the other units combined. These results suggest that overall hand hygiene activity was lower in the ED compared with other units. We also noted that overall glove use in the ED (and in particular in the intervention of direct gloving) was lower. We observed only 7 instances in the ED with direct gloving during which HCP wore gloves on entry to a contact precautions room, suggesting that the intervention (a policy to allow for direct gloving) likely had little effect on the overall practice when glove use and adherence to contact precautions was so low. These observations, while not the primary study outcome, may provide insight into why we found higher bacterial counts on gloves of HCP in the ED. These findings should be explored further in additional ED-based studies. In the meantime, applying a direct-gloving strategy should be guarded, and applying such a strategy in the ED setting or in other areas where overall adherence to hand hygiene and glove expectations are low should be avoided.

Several other factors were associated with detection of pathogens and bioburden on gloved hands. Compared with nurses, physicians had less bacterial contamination on their gloves, whereas other HCP (eg, respiratory technicians, physical therapists) had more bacterial contamination. We also found that a longer time between the last hand hygiene event and sampling was associated with more frequent detection of contamination and higher total colony counts; also, rates of contamination increased with increasing time interval between the last hand hygiene event and sampling. These findings suggest that overall hand hygiene rates at opportunities other than those before glove donning are important for overall bacterial contamination of HCPs’ hands and their gloves.

### Limitations

This study has limitations. The study was completed before the COVID-19 pandemic, and practices may be different during vs after the pandemic. Our study did not have clinical outcomes; however, such a study would be expensive and difficult to adequately power.^20^

## Conclusions

This cluster randomized clinical trial demonstrated that a policy endorsing a direct-gloving strategy led to improved adherence with expected practices and increased overall glove use, was accepted by HCP, and was as safe as the current strategy requiring hand hygiene before donning gloves in areas in which hand hygiene rates were otherwise high. However, a direct-gloving strategy should not be used in the ED or in other areas in which overall adherence to hand hygiene and glove use is low until further studies have been completed.

## Notes

Supplement 1.
Trial Protocol

Supplement 2. **eTable.** Healthcare Personnel Attitudes and Perceptions of a Direct Gloving Strategy

Supplement 3.
Data Sharing Statement
