# Supplementary material for: Direct Gloving vs Hand Hygiene Before Donning Gloves in Adherence to Hospital Infection Control Practices: A Cluster Randomized Clinical Trial
Source: JAMA Netw Open. 2023 Oct 26;6(10):e2336758. doi: 10.1001/jamanetworkopen.2023.36758 (PMC10603500; doi:10.1001/jamanetworkopen.2023.36758)
Supplement: Supplement 1. — Trial Protocol [file jamanetwopen-e2336758-s001.pdf]

PROTOCOL NAME
Efficacy of a direct gloving strategy to improve compliance with infection prevention practices: A cluster-randomized trial (Aim 1)

CICERO protocol number: HP-65259

Modification #: 0

PROTOCOL VERSION DATE: 10/13/2015

Commonly Used Abbreviations

HH	HH
HCW	Healthcare Worker
UMMC	University of Maryland Medical Center
STC	R Adams Cowley Shock Trauma Center
UIH	University of Iowa Hospitals
JHH	Johns Hopkins Hospital
WHO	World Health Organization
ICU	Intensive Care Unit

GENERAL INFORMATION

Name and address of the study monitor and person authorized to sign the protocol and amendments:

Kerri A. Thom, M.D., M.S.
Associate Professor
Division of Genomic Epidemiology and Clinical Outcomes
Department of Epidemiology and Public Health
University of Maryland School of Medicine
Associate Hospital Epidemiologist
University of Maryland Medical Center
685 West Baltimore Street, MSTF 334B
Baltimore, MD, 21201
Phone (410) 706-6124
Fax (410) 706-0098
E-mail kthom@epi.umaryland.edu

UMB Research team
Gwen Robinson (Point of Contact)
Shirley Goodman
Jennifer Johnson
Lisa Pineles
Deborah Stein
Ronald Rabinowitz

Name and addresses of the study sites and/or other institutions involved in the study:

Clinical Sites-

University of Maryland Medical Center
22 S. Green Street
Baltimore, MD 21201

University of Maryland Medical Center
R Adams Cowley Shock Trauma Center
22 S. Green Street
Baltimore, MD 21201

Johns Hopkins Hospital
1800 Orleans St.
Baltimore, MD 21287

The University of Iowa Hospitals
200 Hawkins Dr.
Iowa City, IA 52242

Research offices-
University of Maryland School of Medicine
10 S. Pine St., MSTF 334B, 360
Baltimore, MD 21201

Research Site	Site PI	Point of Contact	Initial IRB Approval	IRB renewal
UMB	Thom, K	Robinson, G	Pending	N/A
UI	Perencevich, E	Cobb, S	Pending	N/A
JHU	Maragakis, L	Rock, C	Pending	N/A

Table of Contents

SUMMARY……………………………………………………………………………4

1. BACKGROUND INFORMATION AND RATIONALE………….…………………………5

1.1. BACKGROUND INFORMATION…………………………………..……..…...……………...5
1.2. RATIONALE…………………………………………………………………..………......5
1.3. RISKS…………………………………………...………………………………...…...6
1.4. BENEFITS………………………………………………………………..………...…..6
1.5. RISK BENEFITS RATIO…………………………………...…………………………..6

2. STUDY OBJECTIVES……………………………………………………………6

3. STUDY DESIGN AND PROCEDURES………………………………………………6

4. SELECTION AND WITHDRAWAL OF PARTICIPANTS………………..……….8

4.1. SELECTION OF STUDY POPULATION……………………………...….…………...8
4.2. INCLUSION CRITERIA……………………………………………………….……………...8
4.3. EXCLUSION CRITERIA…………………………………………………....…...……..8
4.4. PARTICIPANT WITHDRAWAL………………………………………..……...………..8

5. INFORMED CONSENT PROCESS……………………………………………..…8

5.1. RECRUITMENT………………………………………………………………………..8
5.2. ALTERATION OF CONSENT…………………………..………..…..……..……………..9
5.3. WAIVER OF HIPAA AUTHORIZATION………………………………...…...………9
5.4. INFORMED CONSENT PROCESS……………………..……………...…„……...………..9

6. STATISTICAL PLAN……………………………………………………………..10

6.1. ANALYSIS PLAN………………………………...…………………...…………………10
6.2. SAMPLE SIZE ESTIMATES………………...…………...……………………..………………11

7. DATA COLLECTION, HANDLING, AND STORAGE…………………………..12

Summary

This study seeks to investigate alternative strategies to hand hygiene (HH) and glove use in situations where glove use is required to perform health care activities in an effort to increase compliance with infection prevention efforts.

HH is the cornerstone of infection prevention. Despite the importance of and increased focus on HH, compliance remains low in healthcare settings (40% on average in a large meta-analysis). Insufficient time, high workload and under staffing are important barriers. Glove use, which is common and increasing, is another major barrier. New strategies are needed that improve time and efficiency particularly in settings where glove use is required (e.g. Contact Precautions). One area for further study is the requirement for HH prior to non-sterile glove use. This is a recommended practice with poor compliance that may be unnecessary. Furthermore, it may lead to reduced compliance with other recommended infection prevention practices, such as glove use. In this proposal we identify a novel strategy of directly gloving without performing HH prior to non-sterile glove use as a potential solution.

In this study we aim to perform a multicenter, cluster-randomized trial to evaluate the efficacy of direct gloving to improve compliance with infection prevention practices (i.e. HH and glove use). Herein, we will evaluate the safety and efficacy of directly gloving (compared to performing HH prior to glove use) and assess whether this strategy will lead to increased compliance with both HH and glove use. We previously demonstrated the safety of this strategy in a single-center randomized controlled pilot trial where we found no difference in bacterial contamination of gloves of healthcare providers who either performed or did not perform HH prior to donning non-sterile gloves. Thus, with potentially no added benefit and in a setting where we know that HH compliance is the lowest (i.e. prior to glove use), mandating HH prior to donning gloves as recommended in current guidelines could actually reduce both HH and glove compliance, placing patients at increased risk for developing infection.

Hypothesis 1: When compared to the standard practice of performing HH and then donning gloves, directly gloving when non-sterile gloves are indicated will increase compliance with HH and glove use and will have no effect on bacterial contamination of gloves (i.e. total bacterial colony counts).
Aim 1a: Perform a multicenter, cluster-randomized trial to evaluate the efficacy of a direct gloving strategy to improve compliance with infection prevention practices (i.e. HH and glove use).
Aim 1b: Perform a nested multicenter validation study, where gloved hands of healthcare providers will be randomly sampled to determine bacterial contamination of non-sterile gloves after donning.

1 Background Information and Rationale

1.1 Background Information

HH is the cornerstone of infection prevention. Despite the importance of and increased focus on HH, compliance remains low in healthcare settings (40% on average in a large meta-analysis). Insufficient time, high workload and under staffing are important barriers. Glove use, which is common and increasing, is another major barrier. New strategies are needed that improve time and efficiency particularly in settings where glove use is required (e.g. Contact Precautions).

One area for further study is the requirement for HH prior to non-sterile glove use. This is a recommended practice with poor compliance that may be unnecessary. Furthermore, because of the additional time required to cleanse hands and then don gloves, as well as the cumbersome nature of

applying gloves to recently cleansed hands, this practice leads to non-compliance with both HH and glove use – placing patients at risk. A recent randomized trial of 230 health care workers and demonstrated no difference in total bacterial colony counts or identification of pathogenic bacteria from the gloves of persons who either performed HH or did not perform HH prior to putting on non-sterile gloves (Thom, PI, senior author). If unnecessary, HH before non-sterile glove use wastes valuable time, which might otherwise be spent engaged in direct patient care. And removing this unnecessary step may lead to increased compliance with infection prevention measures.

Existing guidelines are confusing and in some places contradictory and all support the need for further research to determine if HH before donning non-sterile gloves is a necessary step. The CDC supports the 2009 WHO HH Guidelines outlining expectations for appropriate HH. In general, the WHO teaches "Five Moments" of HH and uses this model to educate health care workers (HCW) on when HH should occur. This model is also used in surveillance when monitoring and reporting compliance with HH. The *WHO Glove Use Information Leaflet* (2009) under Part II, Section 7 - 'Glove use and the need for HH ' states: "When an indication for HH precedes a contact that also requires glove usage, hand rubbing or hand washing should be performed *before donning gloves*". Yet later in Section 23.1.3, it says: "Whether HH should be performed before donning non-sterile gloves ***is an unresolved issue*** and therefore this moment should not be recommended as an indication for HH ". Furthermore, the question "should HH be recommended before donning non-sterile gloves?" is highlighted within the WHO 'HH Research Agenda' Section.

1.2 Rationale

The necessity of HH before donning non-sterile gloves is unknown. Furthermore, because of the additional time required to cleanse hands and then don gloves, as well as the cumbersome nature of applying gloves to recently washed hands, this practice leads to non-compliance with both HH and glove use – placing patients at risk. As part of our pilot data for this grant, we performed a randomized trial of 230 HCWs and demonstrated **no difference** in total bacterial colony counts or identification of pathogenic bacteria from the gloves of persons who either performed HH or did not perform HH prior to putting on non-sterile gloves. If unnecessary, HH before non-sterile glove use wastes valuable time, which might otherwise be spent engaged in direct patient care. And removing this unnecessary step may lead to increased compliance with infection prevention measures. Despite the lack of supporting evidence, the WHO recommends that healthcare personnel perform HH before putting on non-sterile gloves and most hospital epidemiology and infection prevention groups routinely recommend this practice. Without this study, these non-evidenced based guidelines will persist as a barrier to HH and glove use leading to decreased compliance.

In Aim 1B, we will validate the findings shown by Dr. Thom (PI) et al. demonstrating **no difference** in total bacterial colony counts or identification of pathogenic bacteria from the gloves of persons who either performed HH or did not perform HH prior to putting on non-sterile gloves. Although the findings from the pilot study demonstrate the safety of this alternative strategy, these findings need to be replicated across multiple facilities and varying patient care areas (e.g. pediatrics, emergency departments). Further validation of the pilot findings, as well as results of Aim 1a, will demonstrate generalizability and support guideline/policy change and implementation.

REFERENCES:

1. Centers for Disease Control and Prevention. Guideline for Hand Hygiene in Health-Care Settings: Recommendations of the Healthcare Infection Control Practices Advisory Committee and the HICPAC/SHEA/APIC/IDSA Hand Hygiene Task Force. . *MMWR.* Oct 25 2002;51(RR-16):1-45.

2. WHO Guidelines on Hand Hygiene in Health Care. 2009; http://apps.who.int/iris/bitstream/10665/44102/1/9789241597906_eng.pdf.
3. Rock C, Harris AD, Reich NG, Johnson JK, Thom KA. Is hand hygiene before putting on nonsterile gloves in the intensive care unit a waste of health care worker time?--a randomized controlled trial. *Am J Infect Control.* Nov 2013;41(11):994-996.

1.3 Risks

Aim 1A: The research risks for this study are minimal. In this study, hospital units/wards will be recruited and randomized to either usual care or intervention; no individual patients or HCWs will be enrolled in this study. Preliminary data has demonstrated the safety of a direct gloving strategy (intervention). We hypothesize that by implementing an evidence-based strategy of directly gloving when hand hygiene is indicated will result in an overall improved compliance with infection prevention strategies of hand hygiene and glove use, thus minimizing risks and maximizing benefits. There is a possibility that hand hygiene may decrease in other situations, thus we will measure and monitor hand hygiene compliance at room entry and exit regardless of glove use. All data collected will be for the unit in aggregate and no HCW or patient identifying information will be collected.

Aim 1B: The risks for this aim of study will be minimal for study participants. Study participants are being asked to imprint their gloved hand on an agar plate after donning gloves during routine room entry. The predominant risk to participants in this study is the time required to participate, which will be less than 1 minute. HCWs will be instructed that they can decline participation without repercussion. No identifiable information will be collected from HCW participants. We will not be able to link HCW to individual HCW-patient interactions. Thus, the risk of a breach of confidentiality or privacy is very minimal.

1.4 Benefits

All participating units and their patients, regardless of randomization assignment, stand to benefit from the education regarding opportunities for hand hygiene and other infection prevention practices. This education may lead to an increase in compliance with these practices and potentially may limit the spread of microorganisms to susceptible patients leading to decreased infection.

For Aim 1b, there will be no direct benefit to the HCW participants involved. They will benefit indirectly by knowing that they are contributing to a topic that affects them. The proposed study could lead to changes in policy regarding hand hygiene expectations, monitoring and feedback.

1.5 Risk Benefit Ratio

The potential benefits of this study outweigh the minimal risks to participants.

2 Study Objectives, Aims and Hypothesis

Aim 1A: Perform a multicenter, cluster randomized trial to evaluate the efficacy of a direct gloving strategy to improve compliance with infection prevention practices (i.e. HH and glove use). *Hypothesis:* When compared to the standard practice of performing HH and then donning gloves, directly gloving when gloves are indicated will increase compliance with HH and glove use.

Aim 1B: Perform a nested multicenter validation study, where gloved hands of HCWs will be randomly sampled to determine bacterial contamination of non-sterile gloves after donning. *Hypothesis*: When compared to the standard practice directly gloving when gloves are indicated will have no effect on bacterial contamination of gloves.

3 Study Design and Procedure

Aim 1A:

Study Design: We will perform a cluster-randomized trial to include 14 patient care areas (i.e. units) at the University of Maryland Medical Center (UMMC), the R Adams Cowley Shock Trauma Center (STC), University of Iowa Hospitals (UIH), and Johns Hopkins Hospital (JHH). Each unit will be randomly assigned to the intervention or usual care group. Randomization will be stratified by unit type: adult intensive care, general pediatric wards, emergency departments and inpatient hemodialysis units. One unit from each of the four types at UMMC, UIH, and JHH will be selected for study participation and will be randomized; and two units (one Adult ICU and the Trauma Resuscitation Unit (comparable to the Adult ED)) will be selected and randomized from Shock Trauma. We have specifically chosen these diverse areas to avoid any potential crossover of effect from one study unit to another as units have very different personnel with very little crossover of care.

In order to account for differences in baseline compliance with infection control practices including HH and glove use, baseline rates of compliance will be observed across all available units (this includes 4 adult ICUs, 3 general pediatric wards units, 1 ED and 1 inpatient HD unit at UMMC and 4 adult ICUs and 1 ED at Shock Trauma). In the study period prior to randomization; the final units will be selected based on similar baseline compliance. For example, there are 11 general pediatric wards across three sites. Baseline data will be collected and compared across all 11 wards. One pediatric unit from each facility with the closest baseline composite HH and glove compliance rates will be selected. Thus we are matching 3 general pediatric wards (one from each site) based on pre-intervention outcomes of interest to improve power and minimize the impact of measured and unmeasured confounders. Once selected, the units will be randomized to the intervention or usual care group. This process will be done for each unit type (e.g. adult ICU).

The study will begin with a 6-month period of baseline data collection in which HH and infection prevention compliance will be recorded in all units that are potentially available for inclusion as described above. During that time period the investigative team will develop and refine educational, training and data collection tools. Following this baseline period, a short “wash-in” period of 3 months will be used to educate and train HCWs in the participating units regarding expected infection prevention practices of HH and glove use. The intervention period will be a full 12 months. This will allow the intervention to be tested over a full calendar year accounting for differences in seasonality as well as different levels of training across an academic year.

All participating units regardless of intervention status will receive education regarding policies and procedures for compliance with infection prevention strategies including HH and glove/gown use. This education will be identical in both groups (intervention and usual care) and will be consistent with published guidelines (WHO/CDC) with the exception of HH prior to donning non-sterile gloves - which according to existing guidelines is an uncertain area where research is needed. HCWs from units randomized to the usual care group will receive education that states that they should perform HH prior to donning non-sterile gloves. HCWs from units randomized to the

intervention group will receive education that states that HH is not necessary prior to unsterile glove use. Both units will receive education around other necessary moments for HH and as stated this education will be identical for both groups.

Data collection in both the baseline and intervention phases will be as follows:

Primary Outcome:
Composite Compliance with expected infection prevention practices upon entry to Contact Precaution-patient rooms. HCWs in the usual care group will be recorded as compliant if HH AND glove use is observed at room entry; HCWs in the intervention group only need to have glove use observed to be considered compliant.

Secondary Outcomes:
1. Compliance with glove use upon entry to Contact Precaution-patient rooms for both groups
2. Compliance with HH upon entry to ALL patient rooms
3. Compliance with HH upon exit to ALL patient rooms

Additional Information collected will include: date, time and location (i.e. hospital/unit) of observation and the role of the HCW observed (e.g. MD, RN, other).

Note that all data collected is completely anonymous and no HCW identifying information will be collected nor reported

Aim 1B:

We will perform a nested, multicenter validation study in which we will randomly sample the gloved hands of HCWs entering Contact Precaution-patient rooms in both the intervention and usual care units. Sampling will occur at random and HCWs will not be told in advance that sampling will be performed. A research team member will approach potential participants immediately after gloves are donned and just prior to entering a Contact Precaution-patient room. HCWs will be asked if they have participated in this study previously and those that answer yes will be excluded from participation. Gloved hands of willing HCWs will be sampled via direct imprint of the non-dominant gloved hand onto an agar plate. In addition to sampling of gloved hand, additional data will be collected regarding the date, time and location of the observation, whether the unit was allocated to intervention of usual care, whether the HCW performed HH prior to donning non-sterile gloves, the HCW role (e.g. RN, MD, etc…), when the HCW had started their shift and the time since last performing HH. Each sample will be given a unique number, however, there will be no link to HCW participants and no identifying information will be collected. After sampling, agar plates will be assessed for microbial content including total colony counts and identification of specific pathogenic bacteria including *Staphylococcus aureus*, *Enterococcus*, *Enterobacteriaceae*, *Pseudomonas aeruginosa* and *Acinetobacter baumannii*.

4 Selection and Withdrawal of Participants

4.1 Selection of Study population

Aim 1A. No individual HCW or patient participants will be enrolled in this study. In this cluster-randomized trial, hospital units will be selected for participation and randomized to either the intervention (direct gloving) or usual care strategies. Potential units for participation will include Adult ICU, General Pediatrics, Adult Emergency Department and Inpatient Hemodialysis Units at

the four participating hospitals (University of Maryland Medical Center, RA Cowely Shock Trauma Hospital, University of Iowa Hospitals and The Johns Hopkins Hospital). As outlined above, among the 43 possible units meeting this description, units will be selected for participation based on the results of baseline hand hygiene and glove compliance. 14 total units will be selected for study inclusion and will be randomized to either intervention or usual care. In each case, hospital leadership, unit leadership and the department of infection prevention will assent to unit participation. HCWs in each unit will be observed regarding compliance with hand hygiene and glove use; this data will be collected in aggregate for each unit and no individual healthcare worker data will be collected. This data is currently being collected at each facility as part of standard infection prevention practice.

Aim 1B. Individual HCWs from the above participating units will be randomly selected for participation. Individuals who consent will have samples taken of gloved hands after donning as outlined above.

4.2 Inclusion Criteria

Aim 1B:

- Adults > 18 years
- HCWs at one of the study sites
- HCW donned non-sterile gloves upon entry to a Contact Precaution-patient room

4.3 Exclusion Criteria

- Informed Consent not obtained
- Food services employee
- Environmental services (housekeeping) employee

4.4 Participant Withdrawal

Participants will be unable to withdraw from the study since any data or samples collected will be de-identified. Those who do not wish to participate with sampling of gloves will be able to do so without repercussions for declining participation.

5 Informed Consent Process

5.1 Recruitment

Aim 1A. Ward/units will be recruited for participation in this study. This study aims to recruit from the following unit types at the UMMC: Adult ICUs, General Pediatric Wards, Emergency Departments (ED, TRU) and Inpatient Hemodialysis units. Consideration for a unit/wards participation in this study will be reviewed with the UMMC Department of Infection Prevention and Hospital Epidemiology. In addition, decisions for participation will be made in concert with Infection Prevention AND the individual unit leadership (e.g. Medical and Nursing directors). In the baseline period (previously described), compliance with HH and gown/glove use will be collected and units with similar rates of compliance (across all three sites, U Maryland, Iowa and Hopkins) will be included as study participants and will be randomized to receive the intervention or usual care. Interactions with the participating units will include education and data collection regarding compliance with infection prevention practices.

<u>*Aim 1B*</u>. The investigators will station themselves outside the rooms of patients on Contact Precautions within the units participating in the study; these rooms will be identified by the hospital infection control signs outside the door. As HCWs approach the room with intent to enter; the investigators will introduce themselves, briefly describe the study, study procedures, and will ask HCWs if they would consent to participate. It will be made clear that participation is voluntary and that there are no repercussions for declining participation. The investigators will give verbal information about the study and have written information also available. HCWs will have gloves sampled by direct sampling by agar plate.

5.2 Alteration of Consent

<u>*Aim 1A*</u>:
We have requested an exemption of consent since no individuals (i.e. no human subjects) are being enrolled in the study and data collection is occurring at the aggregate (unit) level - we are not collecting data regarding individual patients or HCWs.

<u>*Aim1B*</u>:
We are requesting a waiver of <u>written documentation of informed consent</u> for participants in this study. HCWs will be approached regarding participation upon entry to patient rooms. Trained research staff will verbally describe the study procedures, requirements, benefits and risks to the subject. The subject will have ample time to ask questions and consider if they want to participate in the study. The subject will provide verbal consent before any procedures are done specifically for the study. The subject may withdraw consent at any point in the study. We are requesting a waiver of written documentation of informed consent - as this might result in greater risk to the participant because it would require that we collect identifying information that we otherwise do not need.

5.3 Waiver of HIPAA Authorization

We are not collecting PHI.

5.4 Informed Consent Process

Aim 1A:
We have requested an exemption of consent since no individuals (i.e. no human subjects) are being enrolled in the study and data collection is occurring at the aggregate (unit) level - we are not collecting data regarding individual patients or HCWs.

<u>*Aim1B*</u>:
This minimal risk study involves direct observation of HH and in randomly selected interactions culturing of gloves by direct imprint on agar plates. It is not scientifically necessary for the study to collect any identifying information about the HCW. By obtaining written informed consent we would need to collect identifiers from participants and thus would place participants at greater than needed risk based on confidentiality. A waiver of written documentation of informed consent has been recommended previously from the IRB on prior similar protocols (ie- culturing skin or attire of HCWs)

6 Statistical Plan

6.1 Analysis Plan

<u>*Aim 1A:*</u>

We will assess whether primary and secondary outcomes are significantly different in the experimental and usual care groups using the generalized estimating equations (GEE) approach with an unstructured working correlation matrix to adjust for clustering. We will perform multivariate analyses using GEE adjusting for covariates such as baseline compliance with HH and frequency of Contact Precautions. Analyses will be conducted at the level of the participating unit and will follow the intention to treat approach.

<u>Aim 1B</u>:

The primary analysis will be an intention-to-treat analysis where the gloved hands of HCWs sampled in the usual care group will be compared to those in the Intervention group regardless of HH activity. A secondary, as-treated analysis will be performed where HCWs who were observed to perform HH and then don gloves will be compared to those in which gloves were directly applied without performing HH. The primary, intention-to-treat analysis will provide great insight into the effectiveness of this intervention while the secondary, as-treated analysis will validate efficacy.

The primary outcome, total bacterial colony forming units (CFUs) > 30 of gloved hands, will be compared between the two groups using a comparison of proportions. In this case a one-sided p <0.025 will be statistically significant for noninferiority. We will also compute a two-sided 95% confidence interval for the relative risk of CFUs > 30 comparing direct gloving to HH + gloving, where the upper bound of the confidence interval < delta will be considered statistically significant. In this analysis, we define the non-inferiority margin, or delta, equal to 1.15. We feel (in consultation with microbiologists and HH experts) that if glove reuse (intervention) were found non-interior to usual care by a 1.15 margin (i.e. a difference of 15%) that this would be enough to lead to change in practice. Robust empirical standard errors will be calculated using generalized estimating equations with an independence working correlation matrix to account for within-site clustering for sample proportions. A secondary analysis will compare groups using CFUs as a count outcome. We will compute a two-sided 95% confidence interval for the relative mean of CFUs on gloved hands comparing the intervention to the usual care group. The upper bound of the confidence interval < delta will be considered statistically significant, where delta is 1.15. The secondary outcomes, presence of pathogenic bacteria and total number of pathogenic and non-pathogenic bacteria present, will be analyzed like the primary outcome.

6.2 Sample Size Estimates

<u>Aim 1A:</u>

How many participants (or specimens, or charts) will be used in this study?

UMMC: 6 units   Worldwide: 14 units

14 units will be randomized to intervention or usual care. Over the 12-month study period we expect a minimum of 7000 total observations (14 units across the systems x 500 observations). Each system will have a research coordinator perform observations 3 hours per day four days per week. We estimate an average of 5 observations per hour[19] (15 per day and 60 per week) and thus over 2500 observations per year per research coordinator are feasible (we expect to require fewer to achieve desired power). Average HH compliance is consistently at 40-50% and are often lower when glove use is indicated[1,15], we believe the compliance with infection prevention practices (i.e. primary outcome, composite compliance with HH AND glove use) in the usual care group will be ~ 40%. Recent studies have shown compliance with gloves when indicated to be approximately 70-80%[15]. Thus for the primary outcome, we expect a difference in compliance with infection prevention practice to be ~ 30%. If we assume 500 observations per area, an alpha level of 0.05, an interclass coefficient of 0.001[22] we will require inclusion of 4 patient care areas (2 experimental and

2 usual care) to achieve a power greater than 90% power to detect an effect size of 30% (i.e. the primary outcome). If we vary the interclass coefficient to 0.01, we still have greater than 90% power under the same conditions. Relative to the secondary outcome, in which we will compare compliance with gloving only in both the direct gloving (i.e. experimental group) and the usual care group, we believe it is reasonable to expect that removing the cumbersome and time-consuming requirement of HH prior to glove use may increase compliance with glove use when indicated by approximately 10%. Using the same assumptions as the primary aim with an interclass coefficient of 0.001, we need to recruit and randomize 4 participating units to achieve > 90% power to detect this effect size of 10%. If we use the higher interclass coefficient of 0.01, we need 8 participating units to achieve > 85% power. So in short, we have ample power to see an effect in both primary and secondary outcomes including the sub-populations of adult ICU, dialysis and emergency department.

Aim 1B:

How many participants (or specimens, or charts) will be used in this study?

Local: 866 Worldwide: 2600

Based on pilot data, we anticipate 5% of HCWs in the usual care group (hand-hygiene plus gloves) and 3.5% in the experimental group (direct gloving) to have > 30 CFUs. We define non-inferiority as a relative risk < 1.15 of having > 30 CFUs, comparing the experimental group to the usual care group. That is, delta equal to 1.15. Under these assumptions, 1280 samples (per group) are sufficient for a one-sided test with alpha=0.025 to have 80% power to detect noninferiority [23]1280 samples in both groups will result in a total of 2560 samples for the 12-month study period. Spread across the 14 proposed participating units at the 3 healthcare systems (UMMC and STC will share a Research Associate), this is approximately 850 samples per site and 180 samples per participating unit over the 12-month study period. Thus, each site will need to recruit just over 70 HCWs each month to meet the goal sample size. In the pilot study 230 HCWs were recruited and agreed to participate in a single month.

7 Data Collection, Handling and Storage

All data collected will be stored in a relational database in order to digitize the data for analysis. No PHI will be collected. The database will be stored on a shared drive. Data from University of Iowa and Johns Hopkins Hospital will be sent electronically the investigators at the University of Maryland – data from Aim 1A is aggregate unit level data and not-human subjects data; and data from Aim 1B is completely de-identified.
