# Supplementary material for: Direct Gloving vs Hand Hygiene Before Donning Gloves in Adherence to Hospital Infection Control Practices: A Cluster Randomized Clinical Trial
Source: JAMA Netw Open. 2023 Oct 26;6(10):e2336758. doi: 10.1001/jamanetworkopen.2023.36758 (PMC10603500; doi:10.1001/jamanetworkopen.2023.36758)
Supplement: Supplement 2. — eTable. Healthcare Personnel Attitudes and Perceptions of a Direct Gloving Strategy [file jamanetwopen-e2336758-s002.pdf]

Supplementary Online Content

Thom KA, Rock C, Robinson GL, et al. Direct gloving vs hand hygiene before donning gloves in adherence to hospital infection control practices: a cluster randomized clinical trial. *JAMA Netw Open*. 2023;6(10):e2336758. doi:10.1001/jamanetworkopen.2023.36758

eTable. Healthcare Personnel Attitudes and Perceptions of a Direct Gloving Strategy

This supplementary material has been provided by the authors to give readers additional information about their work.

© 2023 Thom KA et al. *JAMA Network Open*.

eTable. Healthcare Personnel Attitudes and Perceptions of a Direct Gloving Strategy

Themes	Exemplar quotes
Perceived benefits of direct gloving	• If it's proven safe and effective, I think anything that's cutting down using excess resources and time, that's always a good thing (Pediatric Nurse, Site 3)<br>• It's always good to use less hand sanitizer, because it dries you out [and] that makes you prone to infections as well, because I think we may overuse it. Uh, cost savings, a little bit of time savings. (MICU Nurse Practitioner, Site 1)<br>• I mean there could be cost saving…I'm not sure how entirely expensive it is, but I guess every little bit counts. (NCCU Nurse, Site 3)<br>• My hands would probably not be as dry. We'd probably have better compliance as well. (SICU Physician, Site 2)<br>• Not having to worry about your hands drying. (Pediatric Nursing Assistant, Site 3)<br>• It may be a little bit quicker. (Hemodialysis Physician, Site 3)<br>• I would think it would increase staff satisfaction. (MICU Nurse, Site 1)
Perceived concerns of direct gloving	• I feel like if they found it to be safe then, I don't have concern about it. But, my personal…I'd still wash my hands. (Pediatric Nursing Assistant, Site 3)<br>• Everybody's forgetful at times so, just you could have forgotten. You could have touched something and then, and not [sanitize], and that could be at the time when you go in and transmit something. (MTCC Nurse, Site 2)<br>• I feel like if anything it would be less--, you're giving people more room to not sanitize. (Emergency Department Nurse, Site 1)<br>• It may cause people to be lax in the areas where they're not supposed to be lax when it comes to hand hygiene, or also just overuse of gloves [when] they didn't need the gloves" (Pediatric Physician, Site 3)<br>• And if you're having to remember two different practices, like it's ok to do it in this situation, but in this situation then you would have to like re-sanitize, then I feel like that's setting you up for more error than just trying to focus on one workflow. (SICU Nurse, Site 2)<br>• I think it's better for the hospital to have one protocol for things, and not all be on different pages. (Pediatric Nurse, Site 3)

© 2023 Thom KA et al. *JAMA Network Open*.
