# Supplementary material for: Direct Gloving vs Hand Hygiene Before Donning Gloves in Adherence to Hospital Infection Control Practices: A Cluster Randomized Clinical Trial
Source: JAMA Netw Open. 2023 Oct 26;6(10):e2336758. doi: 10.1001/jamanetworkopen.2023.36758 (PMC10603500; doi:10.1001/jamanetworkopen.2023.36758)
Supplement: Supplement 3. — Data Sharing Statement [file jamanetwopen-e2336758-s003.pdf]

Data Sharing Statement

Thom. Direct Gloving vs Hand Hygiene Before Donning Gloves in Adherence to Hospital Infection Control Practices. *JAMA Netw Open*. Published October 23, 2023. doi:10.1001/jamanetworkopen.2023.36758

Data
Data available: No
